# Gratitude Affects Inter-Subjective Synchronicity for Cognitive Performance and Autonomic Responsiveness

**DOI:** 10.3389/fpsyg.2021.574983

**Published:** 2021-02-24

**Authors:** Michela Balconi, Giulia Fronda

**Affiliations:** ^1^International Research Center for Cognitive Applied Neuroscience (IrcCAN), Catholic University of the Sacred Heart, Milan, Italy; ^2^Research Unit in Affective and Social Neuroscience, Department of Psychology, Catholic University of the Sacred Heart, Milan, Italy

**Keywords:** gratitude, heart rate, skin conductance level, biofeedback, hyperscanning

## Abstract

Recently, social neurosciences have been interested in the investigation of neurophysiological responses related to the experience of positive emotions, such as gratitude, during social interactions. Specifically, the aim of the present research was to investigate whether gratitude related to gift exchange could favor cooperative behavior and bond construction, by improving behavioral and autonomic responsivity. At this regard, the autonomic synchronization and behavioral performance of 16 friends coupled in dyads were recorded during a joint attentional task. Gift exchange could be occurred either at the beginning or in the middle of the task. For the recording of simultaneous autonomic activity [heart rate (HR) and skin conductance level (SCL)], a hyperscanning biofeedback paradigm was used. Intra-subjective analysis showed an increase in behavioral [accuracy (ACC)] and autonomic responses (HR and SCL) when the gift exchange took place at the beginning of the task rather than in the middle. Moreover, inter-subjective analysis revealed an increase in behavioral performance and greater autonomic synchronization of HR index. The present research, therefore, shows how gratitude and trust experienced following gift exchange can modify participants’ reactions by creating a shared cognition and the adoption of joint strategies.

## Introduction

The act of giving or receiving something can be considered as a moment of interpersonal exchange that leads to the development of important links that strengthen social relations (Mick and Demoss, 1990; Mick and Faure, 1998). Specifically, positive emotions experienced during gift exchange, such as gratitude, turn out to be important in the construction of social ties, improving subjective well-being and prosocial behavior by strengthening interpersonal relationships (McCullough and Tsang, 2004; Penner et al., 2005; Algoe et al., 2008; Froh et al., 2008; Lambert et al., 2010). In particular, the moment of gift exchange, as an act of social interaction, involves the individuals by influencing and modeling their behaviors (Golland et al., 2015), thanks to those basic mechanisms that allow to perceive, imitate, and understand others’ feelings, actions, and intentions (Niedenthal, 2007; Balconi and Bortolotti, 2012, 2013; Balconi and Canavesio, 2014). As demonstrated by previous studies, indeed, when these mirroring mechanisms happen in interacting individuals, an implicit behavioral, neural, and psychophysiological activity attunement occurs (Richardson et al., 2007; Konvalinka et al., 2010, 2011; Dumas et al., 2011; Müller and Lindenberger, 2011; Hasson et al., 2012; Giuliano et al., 2015), leading neuroscientists to consider inter-agent actors as a single complex system (Balconi and Pagani, 2015; Chung et al., 2015; Balconi and Vanutelli, 2017a). Specifically, the body synchronization that is experienced between the inter-agents may be due to the sharing of positive emotional experiences during gift exchange (Chauhan et al., 2008; Balconi and Canavesio, 2013). Emotions, indeed, improve behavioral, cognitive, and affective individuals’ synchronization representing the basis of prosocial behavior (Balconi et al., 2011; Balconi and Bortolotti, 2012; Vanutelli et al., 2016, 2017; Balconi and Vanutelli, 2017a).

An innovative paradigm, called hyperscanning, has been proposed to explore the synchronization that occurs between the interacting individuals during joint actions (Vanutelli et al., 2016, 2017; Balconi and Vanutelli, 2017b; Balconi et al., 2018). According to this recent paradigm, the focus is, therefore, on the recording of individuals’ neurophysiological activity during various interpersonal dynamics (Schilbach et al., 2010). As demonstrated by some studies, indeed, the neural and autonomic responses of two interacting individuals can show a strong synchronization during a significant interpersonal exchange (Levenson and Ruef, 1992). Specifically, simultaneous neural activity can be recorded through the use of different techniques, such as electroencephalography (EEG; Koike et al., 2015; Balconi and Vanutelli 2017a), and neuroimaging techniques, such as functional near-infrared spectroscopy (fNIRS), which permit to observe hemodynamic activity changes in specific brain regions (Balconi and Vanutelli, 2017a). On the other hand, peripheral activity can be recorded through the use of biofeedback (Mirgorodsky et al., 2013). In particular, as consistently shown by previous work on autonomic attunement (Balconi and Vanutelli, 2017a; Vanutelli et al., 2017, 2018), the measurement of peripheral synchrony permits to evaluate the physiological synchronization (PS), defined as the covariation of autonomous measurements between dyads or interacting groups (Butler, 2011), by analyzing their changes throughout the task.

The PS index, therefore, can be used to define the intensity of the interaction between the inter-agent individuals (Hatfield et al., 1994). Moreover, the peripheral activity, considered as a response implemented by the sympathetic nervous system (SNS) to identify “fight-or-flight” responses, can provide information on individuals’ emotional state (Diamond and Otter-Henderson, 2007). It turned out to be correlated to some important social, emotional, and empathic processes (Levenson and Ruef, 1992; Adolphs, 2003) and it gives information related to individuals’ synchrony (Chaspari et al., 2015). For these reasons, autonomic synchronization was observed above all in cooperative behaviors (Vanutelli et al., 2016; Balconi and Vanutelli, 2017a) in significant relational ties, such as parent-child interaction (Barrett, 2006) and in the patient-therapist relationship (Marci et al., 2007). Specifically, information on individuals’ emotional activation (Diamond and Otter-Henderson, 2007; Boucsein, 2012) and the quality of social interactions (Guastello et al., 2006) were provided by electrodermal activity (EDA), or skin conductance level (SCL), that is, a representative index of skin conductivity changes. In addition to EDA, the cardiovascular activity also provides information on the individual’s emotional activation. In fact, it increases mainly while experiencing highly positive or negative emotions, such as happiness, joy, fear, sadness, and anger (Levenson, 1992; Sinha et al., 1992), and varies according to emotional closeness (Konvalinka et al., 2011).

These indices, therefore, can provide information related to the emotional synchronization between two individuals involved in social exchange, as revealed in previous studies that observed a higher autonomic activity [increased arousal, SCL, and heart rate (HR)] during social interactions, such as cooperative conditions (Balconi and Bortolotti, 2012). Furthermore, it was shown that the emotional attunement between inter-agents that is experienced during cooperative behaviors leads individuals to improve their behavioral performance through the releasing of dopamine in the prefrontal brain regions. Such release produces an improvement in cognitive performance on a wide range of tasks involving the use of attention and working memory (Gray, 2004; Isen, 2009; Nadler et al., 2010).

The aim of this study was, therefore, to explore the peripheral activity synchronization between two individuals, using a hyperscanning technique based on biofeedback, during the performance of a task that involved a gift exchange (consisting of a material or experiential gift) at the beginning or in the middle of the performance of a cognitive task consisting of three blocks in which was asked to recognize, among others, a target stimulus. Specifically, we hypothesized that the performance of joint action, involving the establishment of cooperative behavior, would lead to an increase in individuals’ peripheral synchronization and behavioral performance. In this regard, an attentive task was devised that required pairs of subjects to cooperate by synchronizing their responses. In particular, the main aim of the present work was to investigate whether the participants’ behavioral performance and peripheral activity improved following the gift exchange. Secondly, we aimed at verifying if the specific moment of gift exchange (at the beginning or in the middle of the task) had specific effects on individuals’ peripheral activity and behavioral responses. Thirdly, we hypothesized that the moment of gift exchange, involving the sharing of positive emotions, such as gratitude, could strengthen individuals’ behavioral performance and autonomic synchronization. Specifically, we expected this effect to be higher in the first condition in which the gift exchange took place at the beginning of the task. Finally, we expected to find different effects in the autonomic activity of the donor compared to that of the receiver before and after the gift exchange, in terms of a higher emotional engagement and a higher arousal for the donor than receiver (Flynn and Brockner, 2003; Duclos and Barasch, 2014).

## Materials And Methods

### Participants

Sixteen pairs of subjects (*N* = 32, *M* = 23.34; *SD* = 1.23) involved in a friendship relationship were recruited to carry out the experiment, using the following exclusion criteria: normal or correct-normal visual acuity and absence of neurological or psychiatric pathologies, verified by specific measurements. Two dyads were excluded due to the low quality of autonomic signal. The dyads took part in the study after signing the informed consent. The research was conducted according to the Helsinki Declaration and was approved by the local ethics committee of the Department of Psychology of the Catholic University of Milan.

### Procedure

To carry out the experiment, the subjects were seated in a dark room at a 60 cm from a computer.

**Figure 1:**
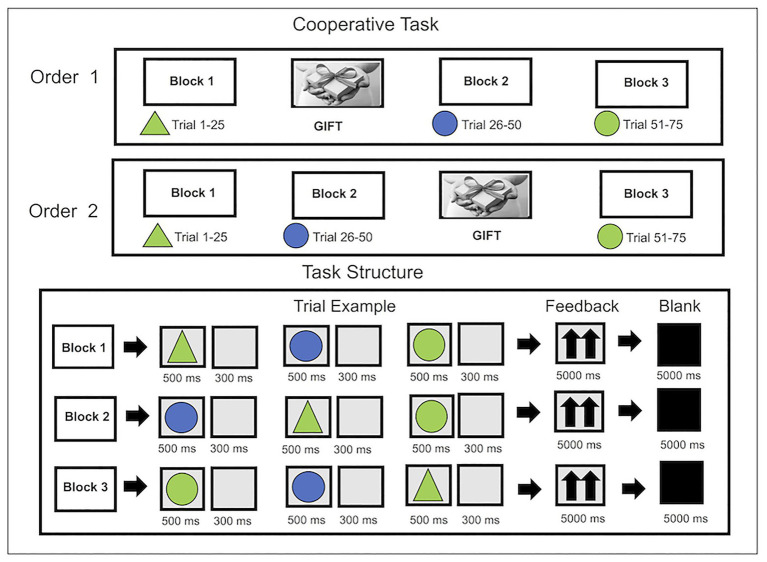
Experimental procedure. Two different procedures were performed: order 1 composed by block 1 (a control condition), gift exchange, and blocks 2 and 3; and order 2 composed by block 1, block 2, gift exchange, and block 3. Blocks 1, 2, and 3 involve a cooperative task which consisted of a game of selective attention.

Specifically, the subjects were asked to carry out a computerized task that involved a gift exchange at the beginning or in the middle of the task, which had to be donated by one of the subjects of the dyad (donor) to his partner (receiver). For seven couples, the gift exchange occurred before the beginning of the first part (after block 1); for the other seven pairs, it occurred at the end of the second block. Two different procedures were used: the first involved the performance of a basic condition (block 1), the gift exchange, and the execution of the other two task blocks (blocks 2 and 3), and the second provided for the unwinding of blocks 1 and 2, the gift exchange, and the carrying out of the block 3. Therefore, at the beginning or in the middle of the task (concerning the two procedural orders), they were asked to exchange a gift, which could be a material or experiential object (objects or tickets to visit a museum or a concert). Before the execution of blocks 2 and 3, after recording a basic condition of 120 s, the participants were asked to perform a familiarization activity with the activity (block 1; Figure 1).

Specifically, the execution of the three blocks required the participants to cooperate during the development of a selective attention task modified by a previous computerized version (Balconi and Pagani, 2015; Balconi and Vanutelli, 2016; Vanutelli et al., 2016, 2017; Balconi et al., 2018, 2019a,b, 2020; Balconi and Fronda, 2020). The task required subjects to memorize a target stimulus (triangle or circle and green or blue) that they should have subsequently recognized among others by pressing the right or left key of the computer keyboard. Specifically, the stimulus appeared on the screen for 500 milliseconds (ms) with an inter-stimulus interval (ISI) of 300 ms and an inter-trial interval (ITI) of 5,000 ms. The task required the subjects to recognize the target stimulus by synchronizing the speed and accuracy (ACC), understood as the percentage of the correct responses concerning the recognition of the target stimulus, of their responses with the support of some feedback indicating the individuals’ cooperation level represented by two arrows pointing upward, indicating a level of cooperation over 75%. Then, the subjects were asked to temporarily associate their reaction times (RTs) to increase their perception and psychological condition of cooperation and joint task. At the end of the task, both participants were given a questionnaire in order to investigate the perception of the partner (his/her friend) and the level of couple tuning during the performance of the first and second task blocks. The questionnaire consisted of the following items: “What was the perception of your workmate in the first phase of the game?,” “What was the perception of your workmate in the second phase of the game?,” “What was the perception of your collaboration and degree of gratitude in the first phase of the game?,” and “What was the perception of your collaboration and the degree of gratitude in the second phase of the game?.” Participants could respond to items by assigning a Likert-scale score from 1 (perception of non-synchronicity/non-cooperation) to 3 (perception of synchrony and cooperation).

### Autonomic Measures Recording and Analysis

The autonomic activity was recorded using two Expert2000 portable Biofeedback systems with a MULTI radio module (Schuhfried GmbH, Mödling, Austria) that allow to measure the level and response of SCL in μS and HR in beats per minute (bpm). The SCL value was recorded with an EDA1 gold electrode using current measurement at a sampling frequency of 2 kiloHertz (kHz). The use of alternating voltage prevents polarization. The measurement resolution for the SCL calculation is 12 nanosecond (ns) with a sampling frequency of 20 Hertz (Hz). HR was measured by the infrared absorption principle with a sampling frequency of 500 Hz. The parameter range was 30–200 bpm. Furthermore, the mobility of the non-dominant hand was monitored with an accelerometer in meter/square second (m/s2) integrated into the sending unit to ensure that the recordings were not compromised by hand movements. Trials with motor artifacts have been removed from the analyses.

## Data Analysis

### Questionnaire Responses

For the answers to the questionnaire, a preliminary analysis was conducted. Specifically, two mixed model ANOVAs with Block (pre vs. post) and Role (donor vs. receiver) as a repeated factor, and Condition as between-subjects factor (Cond: order 1 vs. order 2) were applied to questionnaire scores.

ANOVA results showed a significant effect for Block (*F*[1,97] = 22.56; *p* < 0.0001; *η*^2^ = 0.90), with a greater perception of tuning after (*M* = 3.0; *SD* = 0.07) than before (*M* = 1.04; *SD* = 0.05) gift exchange. With regard to the perception of cooperation, instead, ANOVA showed a significant effect for Block (*F*[1,97] = 34; *p* < 0.0001; *η*^2^ = 0.94), with a greater perception of cooperation later (*M* = 2.99; *DS* = 0.06) compared to before (*M* = 1.34; *SD* = 0.03) gift exchange.

### Behavioral and Autonomic Data Analyses

Three main orders of analyses were performed: (1) Repeated measures ANOVAs on the modulation of the dependent variables (ACC, RTs, SCL, and HR) for the subjects throughout the task were conducted (A – intra-subjective analysis); (2) inter-subjects correlational indices were performed (B – inter-subjective analysis) to compute the synchronization values within each couple for each autonomic measure; and (3) repeated measures ANOVAs on such indices to assess differences in synchrony strength across the experimental conditions. For all the ANOVA tests, the degrees of freedom were corrected using Greenhouse-Geisser epsilon where appropriate, with the level of significance set at 0.05. Also, *post-hoc* comparisons (contrast analyses) were applied to the data. Bonferroni test was applied for multiple comparisons. In addition, the normality of the data distribution was preliminary tested (kurtosis and asymmetry tests). The normality assumption of the distribution was supported by these preliminary tests.

### (A) Intra-Subjective Analysis

#### Behavioral Results

Accuracy and RTs scores were obtained for each subject using E-prime software during the three blocks. Specifically, for the analysis of ACC, the percentage of correct answers on the total answers was considered, while RTs were calculated starting from the presentation of the stimulus.

Before the pre-gift training condition, after the 120-s reference record, the subjects were provided a familiarization task. Specifically, two mixed-model ANOVAs were applied to ACC and RT with Blocks (1baseline vs. 2 vs. 3) as a repeated factor and Condition (Cond: order 1 vs. order 2) and Role (Role: donor vs. receiver) as between-subjects factor.

For ACC, ANOVA revealed a significant effect for Cond (*F*[1,60] = 12.34; *p* < 0.001; *η*^2^ = 0.38), with better performance for order 1 than order 2; Block (*F*[2,97] = 9.77; *p* < 0.001; *η*^2^ = 0.32) and Cond × Block (*F*[2,97] = 12.98; *p* < 0.001; *η*^2^ = 0.37). In particular, as shown by *post-hoc* comparisons applied to interaction effects, order 1 revealed a higher ACC in block 2 (*F*[1,31] = 9.32; *p* < 0.001; *η*^2^ = 0.31) and in block 3 more than block 1 (*F*[1,31] = 8.87; *p* < 0.001; *η*^2^ = 0.29).

**Figure 2:**
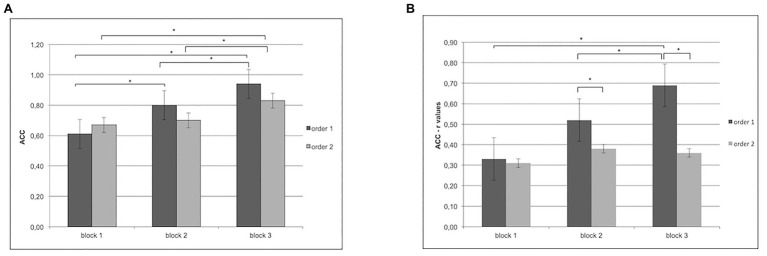
**(A)** Increase in accuracy for order 1 in blocks 2 and 3 more than block 1 and in block 3 more than block 2. For order 2, instead, the figure shows an increase of accuracy in block 3 more than blocks 1 and 2. **(B)** Increase of inter-subjective accuracy (*r* values) for order 1 more than order 2 in block 2 and block 3. Moreover, for order 1, the figure shows an increase of inter-subjective accuracy (*r* values) in block 3 more than block 1. In addition, higher synchronicity was observed in order 1 for block 3 more than block 2.

Moreover, block 3 differed from block 2 (*F*[1,31] = 8.50; *p* < 0.001; *η*^2^ = 0.29) with higher ACC. In contrast, order 2 showed higher ACC only in block 3 more than block 1 (*F*[1,31] = 9.08; *p* < 0.001; *η*^2^ = 0.30) and block 2 (*F*[1,31] = 9.16; *p* < 0.001; *η*^2^ = 0.32; Figure 2A).

No significant effects were found for RTs.

#### Autonomic Measures

**Figure 3:**
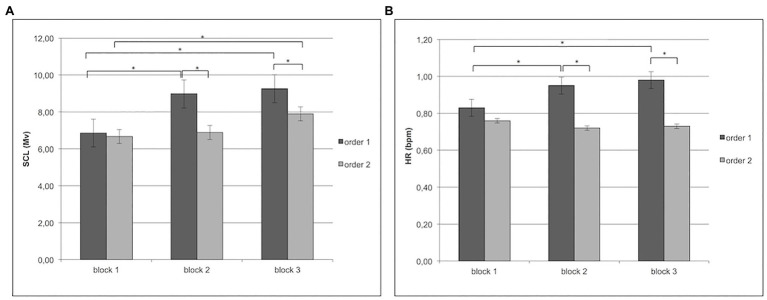
**(A)** Increase in individuals’ skin conductance level (SCL) for order 1 in block 2 and block 3 more than order 2. Moreover, the figure shows for order 1 an increase of SCL in blocks 2 and 3 more than block 1; while, for order 2, an increase of SCL in block 3 more than block 1. **(B)** For heart rate (HR), the figure shows an increase of HR for order 1 more than order 2 in block 2 and block 3. Specifically, for order 1, the figure shows an increase of HR in blocks 2 and 3 more than block 1.

About SCL, Cond × Block interaction effect was significant (*F*[2,97] = 7.94, *p* < 0.001, *η*^2^ = 0.29). Specifically, as revealed by *post-hoc* comparisons, there was an increase of SCL for order 1 more than order 2 in block 2 and block 3 (respectively, *F*[1,31] = 9.56, *p* < 0.01, *η*^2^ = 0.33; *F*[1,31] = 8.99, *p* < 0.01, *η*^2^ = 0.29). In addition, in order 1, block 2 and block 3 differed from block 1 with increased SCL (*F*[1,31] = 8.55, *p* < 0.01, *η*^2^ = 0.28), whereas the differences in order 2 were found only between block 1 and block 3 (respectively, *F*[1,31] = 8.90, *p* < 0.01, *η*^2^ = 0.29; *F*[1,31] = 9.56, *p* < 0.01, *η*^2^ = 0.32; Figure 3A).

About HR, Cond × Block interaction effect was significant (*F*[2,97] = 9.33, *p* < 0.001, *η*^2^ = 0.35). Specifically, as revealed by *post-hoc* comparisons, there was an increase of HR for order 1 more than order 2 in block 2 and block 3 (respectively, *F*[1,31] = 12.09, *p* < 0.01, *η*^2^ = 0.36; *F*[1,31] = 9.09, *p* < 0.01, *η*^2^ = 0.31). In addition, in order 1, block 2 and block 3 differed from block 1 with increased HR (respectively, *F*[1,31] = 10.06, *p* < 0.01, *η*^2^ = 0.37; *F*[1,31] = 9.50, *p* < 0.01, *η*^2^ = 0.34; Figure 3B).

### (B) Inter-Subjective Analyses

#### Calculation of Correlational Indices

The synchronization indices were calculated by correlational coefficients (Pearson coefficients) applied to the data for each behavioral (ACC and RTs) and autonomic index (SCL and HR; Hernandez et al., 2014).

According to these indices, the subsequent step of analysis was finalized to test the statistical significance of independent factor Blocks (1baseline vs. 2 vs. 3) and Condition (Cond: order 1 vs. order 2) on these correlational indices for each couple by using repeated measures ANOVAs.

#### Behavioral Measures

For ACC, ANOVA showed a significant Cond × Block interaction effect (*F*[2,97] = 8.89, *p* < 0.01, *η*^2^ = 0.29). Specifically, pairwise *post-hoc* comparisons revealed a significant higher synchronization (higher *r* values) for order 1 more than order 2 in block 2 and block 3 (respectively, *F*[1,13] = 9.11, *p* < 0.01, *η*^2^ = 0.32; *F*[1,13] = 8.44, *p* < 0.01, *η*^2^ = 0.30). In addition higher synchronicity was observed in order 1 for block 3 more than block 1 (*F*[1,13] = 6.34, *p* < 0.01, *η*^2^ = 0.26) and block 2 (*F*[1,13] = 6.89, *p* < 0.01, *η*^2^ = 0.27; Figure 2B).

No significant effect was found for RTs.

#### Autonomic Measures

For what concerns SCL coefficient data, no significant differences in synchrony were found for the main or interaction effects.

**Figure 4:**
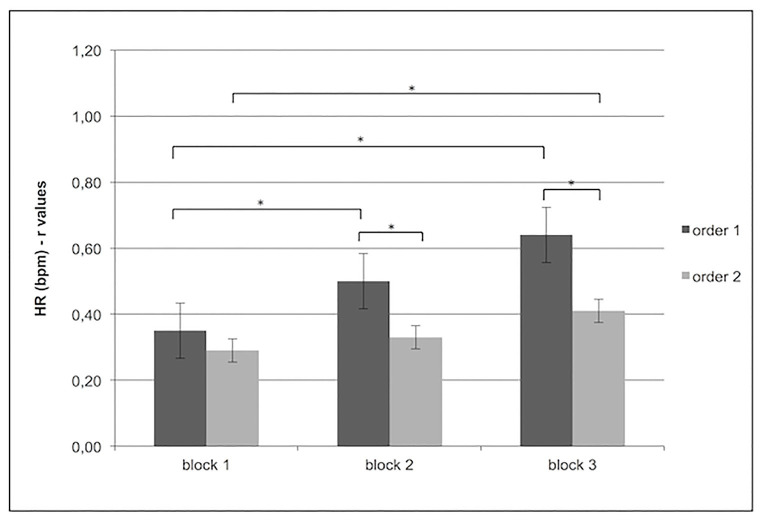
Increased HR subjects’ synchrony (*r* values) for order 1 more than order 2 in block 2 and block 3. Moreover, for order 1, the figure shows an increase in HR synchrony in blocks 2 and 3 more than block 1 and in order 2, an increase of HR subjects’ synchrony was found block 3 more than block 1.

In contrast, significant main effect for Condition × Block was found for HR (*F*[1,15] = 8.87, *p* < 0.01, *η*^2^ = 0.29), which showed increased HR synchrony for order 1 more than order 2 in block 2 (*F*[1,13] = 6.34, *p* < 0.01, *η*^2^ = 0.26) and block 3 (*F*[1,13] = 6.34, *p* < 0.01, *η*^2^ = 0.26). In addition in order 1, increased synchrony was found more in block 2 (*F*[1,13] = 6.34, *p* < 0.01, *η*^2^ = 0.26) and block 3 (*F*[1,13] = 7.09, *p* < 0.01, *η*^2^ = 0.27) than block 1, whereas in order 2, increased synchronicity was found only for block 3 compared to block 1 (*F*[1,13] = 6.34, *p* < 0.01, *η*^2^ = 0.26; Figure 4).

## Discussion

The present research has analyzed a joint action dynamic through the application of a multimethodological paradigm requiring the recording of participants’ autonomic responses and behavioral performance during a cooperative activity involved a beginning or middle gift exchange. In addition to previous studies (Balconi et al., 2019a,b, 2020; Balconi and Fronda, 2020) aimed at detecting central indices (EEG or hemodynamic activity), which allow obtaining different information on different cognitive processes (Herrmann et al., 2003, 2008), the measurement of autonomic activity can be considered as a potential biological marker of emotions (Tupak et al., 2014), which allows to better define the interaction between peripheral and central systems (Furmark et al., 1997; Lang et al., 2000). Data were analyzed both at the individual (intra-subject) and at the dyadic level (inter-subject) to calculate PS and before and after gift exchange. Indeed, we hypothesized that sharing a gift could be accompanied by a positive emotional engagement that could influence both the behavioral and the physiological responses.

The analyses allowed to highlight some main results. (1) An improvement of both behavioral and autonomic responsivity has emerged in order 1, when gift donation came earlier, compared to a delayed exchange. A similar pattern emerged also at the inter-personal level, with increased physiological synchronization after the early gift. (2) The modulation of such effects by block variable was observed. In detail, both at the individual and the joint level, the advantage of order 1 was maximized over the blocks in favor of the last block. (3) Both cardiovascular (HR) and electrodermal (SCL) indices proved to be effective in detecting participants’ individual and joint responses. However, HR was more sensitive to such dynamics, especially at the interpersonal level. Finally, (4) although condition and blocks were able to modulate participants’ responses during the task, the specific role adopted (donor or receiver) was not influential.

Beginning with the first point (1), an earlier gift exchange was associated with increased accuracy and higher autonomic responses in detecting the attentional targets both at the individual level and the interpersonal level. Such result can be interpreted by referring to the construct of gratitude. In fact, giving and receiving a gift within an interpersonal interaction is associated with a positive feedback loop which leads to emotional sharing (Aknin et al., 2011). Previous research, indeed, already underlined the influence of gratitude and positive emotions in strengthening cooperative behavior (Rumble et al., 2010). Such greater tuning was also explicitly attested by the participants through the self-report questionnaire. According to previous research, these emotional states can be the consequence of sharing a pleasant experience, which increases the feeling of being part of a whole and the sense of interpersonal cohesion (Balconi and Pagani, 2015; Chung et al., 2015; Vanutelli et al., 2017). Moreover, such result is of particular importance since this kind of interpersonal attuning makes the implementation of prosocial behaviors more likely (Ruby and Decety, 2004; Spinella, 2005; Balconi et al., 2011; Balconi and Bortolotti, 2012) and represents a social glue thanks to a reciprocity mechanism (Balconi and Lucchiari, 2006; Balconi and Pozzoli, 2007).

However, to better understand our results, it is also important to consider trust mechanisms. Indeed, although both order 1 and order 2 involve gift exchange, order 1 could have facilitated bond construction, emotional sharing, and cooperation, since it immediately allowed participants to increase their trust based on initial gift exchange. Previous research underlined how the experience of trust is highly influenced by mood and emotions (Jones and George, 1998). Accordingly, we believe that an interaction based on trust since its beginning could be more efficient in engaging participants in a cooperative activity. Such result finds support in previous research that showed a relation between bond construction, cooperation, and interpersonal coordination, even in studies with unrelated participants (Chung et al., 2015; Balconi and Vanutelli, 2016; Vanutelli et al., 2017; Balconi et al., 2018). For example, research on autonomic synchrony showed that the covariation between couples’ physiological indices can reveal insights about the quality of their interaction representing a key marker of social engagement (Vanutelli et al., 2017).

This hypothesis is also corroborated by the second result (2). Indeed, the advantage of an earlier gift, shown by significant effects founded across the three blocks is potentially relevant to suppose a trend with an increased synchronization during the task and over the time. Even if order 2 displays an increasing synchronization over blocks, order 1 has a more definite distribution of both behavioral and autonomic responses, which can be appreciated especially between blocks 2 and 3. Indeed, in order 1, the gift is donated after the first block. Thus, there is sufficient time for the participants to develop dyadic strategies that become refined and improved in the last block. Contrarily, in order 2, the gift is donated between blocks 2 and 3, in a way that the effect of positive emotions cannot be translated into consolidated joint strategies both at behavioral and autonomic levels, as shown for order 1.

For what concerns this last point, it is important to underline the functional role of the autonomic responses (3). At the individual level, both SCL and HR proved to be sensitive to gift exchange modulation. However, referring to PS, a systematic synchronization emerged only for HR correlational indices. Interestingly, although SCL can be interpreted in relation to emotional arousal (Sohn et al., 2004), an increase in HR indices has been reported when experiencing highly positive emotional states (Ekman et al., 1983; Levenson, 1992; Sinha et al., 1992). Also, the covariation of such indices within an interpersonal relationship can be an index of emotional closeness (Konvalinka et al., 2010) and trust. Moreover, the degree of HR synchrony could predict participants’ expectations about the moves of their partners (Mitkidis et al., 2015). Since SCL is generally interpreted as an emotional-arousal related measure (Picard et al., 2016), the simple increasing of arousal is not enough to support a synchronous response by the members of the dyad.

Finally, the role played by the two members of the dyad (donor or receiver) did not have any significant effects in modulating participants’ responses. Thus, we could assume that both the act of giving and receiving a gift can similarly contribute to creating stronger cooperative ties, underlining how the implementation of prosocial behaviors can represent a social reward, even without a material return (Vanutelli et al., 2016). However, the absence of significant differences based on the role may be due to the familiarity and previous friendship between donor and receiver.

## Conclusion

To conclude, the present study showed how gratitude and trust elicited within a gift donation protocol can modify the individuals’ behavioral and autonomic responses. The moment of gift exchange can actually influence the creation of a positive emotional feedback loop and the perception of trust. When the gift is donated early, participants have the possibility to build a safe interpersonal space to develop a shared cognition, which provide an increase of performance, autonomic responses, and synchrony. This hypothesis is supported by the fact that this pattern proved to follow an exponential path, which reaches its maximum strength in the last experimental block, and by the specific autonomic index involved.

We believe that our paradigm is innovative and that it could be shared among scholars and applied to different real-life contexts where trust can be improved to provide a better emotional experience, such as the organizational, the educational, and the clinical framework. Some further developments may be proposed: The integration of neurophysiological and neuroimaging measurements would allow obtaining information on the neural individuals’ synchronization and the integration of psychometric measures would allow evaluating some personalities traits; the comparison between dyad of subjects with and without previous friendship to better explore the effect of familiarity in distinguishing the role effect. Finally, the role of gender in cooperation and trust could be explored.

## Data Availability Statement

The raw data supporting the conclusions of this article will be made available by the authors, without undue reservation.

## Ethics Statement

The studies involving human participants were reviewed and approved by local Ethics Committee of the Department of Psychology of the Catholic University of Milan. The patients/participants provided their written informed consent to participate in this study.

## Author Contributions

MB contributed to the conception and design of the study and wrote the first draft and each section of the manuscript. MB and GF contributed to manuscript final writing and revision, and read and approved the submitted version.

### Conflict of Interest

The authors declare that the research was conducted in the absence of any commercial or financial relationships that could be construed as a potential conflict of interest.
